# Transplantation for Congenital Bone Marrow Failure Syndromes

**DOI:** 10.1155/2011/849387

**Published:** 2010-11-29

**Authors:** Kenji Morimoto, Theodore B. Moore, Gary Schiller, Kathleen M. Sakamoto

**Affiliations:** ^1^Division of Hematology/Oncology, Department of Pediatrics, David Geffen School of Medicine at UCLA, University of California, 10833 Le Conte Avenue, Los Angeles, CA 90095-1752, USA; ^2^Division of Hematology/Oncology, Department of Medicine, University of California, Los Angeles, CA, USA; ^3^Gwynne Hazen Cherry Memorial Laboratories and Mattel Children's Hospital, Jonsson Comprehensive Cancer Center, Molecular Biology Institute, California NanoSystems Institute, David Geffen School of Medicine at UCLA, Los Angeles, CA, USA

## Abstract

Congenital bone marrow failure syndromes (BMFSs) are relatively rare disorders characterized by aberrant development in one or more hematopoietic lineages. Genetic alterations have now been identified in most of these disorders although the exact role of the molecular defects has yet to be elucidated. Most of these diseases are successfully managed with supportive care, however, treatment refractoriness and disease progression—often involving malignant transformation—may necessitate curative treatment with hematopoietic stem cell transplantation. Due to the underlying molecular defects, the outcome of transplantation for BMFS may be dramatically different than those associated with transplantation for more common diseases, including leukemia. Given recent improvements in survival and molecular diagnosis of bone marrow failure syndrome patients presenting at adult ages without physical stigmata, it is important for both pediatric and adult hematologists to be aware of the possible diagnosis of BMF syndromes and the unique approaches required in treating such patients.

## 1. Introduction

Bone marrow failure syndromes (BMFSs) are a constellation of disorders characterized by abnormal hematopoiesis and often accompanied by assorted physical findings and a strikingly increased risk for hematologic malignancy. Historically, patients presented with significant cytopenias and physical stigmata during childhood. However, it is now evident that BMFS posses a broad range of phenotype penetrance and an increasing number of patients are being identified in adulthood, occasionally presenting with leukemia or aplastic anemia. Some BMFS disorders are associated with defective DNA repair or maintenance pathways and possess increased sensitivity to DNA-damaging agents including chemotherapy and radiation. These sensitivities have been clearly demonstrated by the significantly increased chemotherapy-related and stem cell transplant-related morbidity and mortality in BMFS patients. It is therefore important for both pediatric and adult hematologists to be aware of the presentation, management, complications, and approaches to treat BMFS. We review here transplantation approaches and outcomes in patients with Fanconi Anemia, Diamond-Blackfan anemia, dyskeratosis congenita, severe congenital neutropenia, and Shwachman-Diamond syndrome to illustrate their relative risks for malignancy and reported utility of varying transplant regimens. The reporting of cumulative transplant outcomes will hopefully add clarity to the indications for transplantation in these disorders.

## 2. Fanconi Anemia

Fanconi Anemia (FA) is a disease of abnormal DNA repair resulting in abnormal hematopoiesis, variable anatomic phenotypes, and an increased predisposition to malignancy. 13 FANC genes have been identified that are responsible for forming an ubiquitinating nuclear core complex, acting as a chromatin binding cofactor for other DNA repair enzymes, or exerting DNA helicase activity all in response to DNA intrastrand cross-linking damage. The gene FANCD1 was previously identified as BRCA2, the breast cancer susceptibility gene 2. 

Physical findings may include short stature and skeletal abnormalities (classically involving radius or thumb), developmental delay, abnormal kidneys, skin pigmentation/café au lait spots, and triangular facies in 60%–75% of patients. Hematologic cytopenias are almost always present at diagnosis and may include uni-, bi-, or trilineage involvement. The risk of early bone marrow failure appears to correlate with the presence of certain physical abnormalities and high-risk patients face a 7% annual incidence of bone marrow failure [1]. Conversely, patients without physical findings or subtle physical abnormalities are at less risk for early bone marrow failure and may thus present in adulthood. Ten percent of FA patients may present at greater than 16 years of age [1]. Fanconi Anemia patients are at increased risk of developing malignancies including acute myeloid leukemia (AML), myelodysplastic syndrome (MDS), head/neck cancers, and gynecologic malignancies; the relative risk of developing specific solid tumors and AML/MDS ranges from several hundred- to several thousand-fold compared to the general population [2, 3]. 

Given the high risk of bone marrow failure and clonal abnormalities, FA patients are often considered for hematopoietic stem cell transplantation. Initial transplantation attempts with antigen-matched donors resulted in 80% mortality at 3 years when conditioned with cyclophosphamide 200 mg/kg and 500 cGy thoraco-abdominal irradiation (TAI) [4]. *In vitro* studies of FA primary cells demonstrated increased sensitivity to cyclophosphamide [5] and irradiation [6]. Subsequent transplants have thus used reduced intensity conditioning regimens and achieved increased survival and decreased graft versus host disease (GVHD) severity. At present, transplant from a sibling-matched donor remains the optimum therapy for FA. A recent series of 30 patients without demonstrable clonality or malignancy receiving sibling-matched donor bone marrows used a conditioning regimen of ATG, cyclophosphamide 20 mg/kg (40 mg/kg for one 5/6 sibling match), 400 cGy TAI (450 cGy for lone 5/6 sibling match), and GVHD prophylaxis of cyclosporine and steroids. The authors reported a 97% neutrophil engraftment rate and 77% five-year overall survival with an incidence of 20% acute GVHD (aGVHD) and 7% chronic GVHD (cGVHD) [7]. 

In contrast, Wagner et al. reported 98 FA patients (78 HLA identical matches) who underwent an unrelated bone marrow transplantation and were conditioned with TBI (450 cGy), cyclophosphamide (20–40 mg/kg), and ATG ± fludarabine. The addition of fludarabine (used in post-1998 transplants) with T-cell depletion significantly enhanced neutrophil engraftment rates (89%), decreased aGVHD Grade II–IV probability (16%), and increased adjusted 100-day survival (76% versus 35%) and 3-year survival (52% versus 13%) compared to nonfludarabine regimens. Overall mortality within the fludarabine-conditioned patients was secondary to graft failure (15%), GVHD (11%), and organ failure (11%) [8]. Gluckman et al. reported the outcome of 93 unrelated umbilical cord stem cell transplant recipients with half of patients receiving a 6/6 or 5/6 matched cord, the other half receiving 2- or 3-HLA mismatched cords. Sixty one percent of patients received fludarabine-based regimens, 80% received TBI, and 81% received anti-T cell therapy. Median average cell count was 1.9 × 10^5^ CD34+ cells/kg and 4.9 × 10^7^ TNC/kg. Neutrophil engraftment at the relatively late time-point of 60 days was achieved by 60% (median time to neutrophil recovery for these patients was 23 days), incidence of acute GVHD Grade II–IV 32% (no aGVHD observed in matched cords), and a 3-year overall survival of 40% (50% in patients receiving fludarabine-based conditioning) [9]. Patients receiving 6/6, 5/6, and 3-4/6 matched cords had 3-year OS of 74%, 48%, and 25% respectively. Of the 56 deaths, mortality was largely due to infection (31%), graft failure (21%), and aGVHD (12.5%).

Acknowledging the increased risks irradiation may pose for FA patients, an international retrospective study of 148 sibling-matched patients receiving irradiation, cyclophosphamide ± ATG versus cyclophosphamide alone or in combination with ATG, fludarabine, or busulfan reported similar neutrophil engraftment rates by day 28 (94% versus 89%, resp.) and day 100 (92% each), similar incidence of Grade II–IV acute GVHD (23% versus 21%, resp.), and similar adjusted probability of 5-year survival (78% versus 81%, resp.) with similar mortality from infection, GVHD, and organ failure [10]. Consistent with these findings, Locatelli et al. analyzed the utility of cyclophosphamide and fludarabine conditioning in 31 sibling-matched HSCT patients with a reported estimated 8-year OS of 87% [11]. A radiation dose reduction study at the University of Minnesota reported 100% engraftment with 300 cGy TBI in 22 FA patients receiving MUD transplants (although both patients receiving 150 cGy experienced secondary graft failure) [12]. 

Transplantation remains the front-line therapy for bone marrow failure in FA patients. Sibling-matched donors have traditionally been the donor although MUD BMT and UCSCT outcomes have improved with the incorporation of fludarabine into the conditioning regimen. Current data support the feasibility of reducing radiation dosages from the standard conditioning regimens in MUD transplants, and perhaps elimination of radiation in sibling-matched transplants.

## 3. Diamond Blackfan Anemia

Diamond Blackfan Anemia (DBA) is a congenital disorder consisting of red cell aplasia and assorted physical abnormalities including short stature, craniofacial abnormalities, and radial irregularities. Consistent with other congenital BMF syndromes, DBA patients have an increased risk of developing malignancies. A longitudinal followup of 72 DBA patients reported 4 cases of AML ranging 15–30 years after the diagnosis of DBA [13]. Osteogenic sarcoma has also been reported in DBA patients [14]. Over 50% of DBA patients possess a mutation in a ribosomal protein, although the exact pathophysiology of the disease remains unclear. Patients typically present in infancy with a macrocytic anemia [15]; erythrocytes have disrupted maturation and possess elevated adenosine deaminase levels [16]. There is a spectrum of anemia severity and red blood cell transfusion requirements. Steroids are the front-line medical therapy and approximately 70%–80% of DBA patients will initially have a transfusion free response [17, 18]. Only 20% retain a sustained remissions off of steroids and thus steroid-related complications and transfusion-induced iron overload remain significant problems. 

Case reports of HSCT in DBA date back to 1976 [19] but large studies are limited. A retrospective review analyzed transplants from 8 sibling matched versus 12 MUD donors in 20 patients, resulting in a 5-year overall survival of 87.5% and 28%, respectively [20] (excluding one death from osteogenic sarcoma in the MUD arm). These results are confounded by differences in radiation containing conditioning regimens. Roy et al. [21] reported IBMTR registry outcomes of 61 transplanted DBA patients (median age 7 years), with two-thirds receiving transplants from matched sibling donors and 20% from MUD (remainder received nonsibling family donor). 95% of sibling-matched recipients received myeloablative, cyclophosphamide-based conditioning, and 45% of MUD recipients received TBI-based conditioning. All patients received bone marrow stem cells. GVHD prophylaxis consisted of cyclosporine, methotrexate, or both in 90% of the patients. Ninety-two percent and 75% of all patients demonstrated neutrophil and platelet engraftment by Day 100, respectively with no difference between the two arms (although Day 28 engraftment rates were significantly higher in the sibling-matched arm). Total nonengraftment incidence was 9%. Cumulative incidence of acute GVHD grade II–IV was 28% and cGVHD was 19% with overall incidence being too small to detect differences between the two donor types. Heavily transfused patients (receiving greater than 50 transfusions) had slower engraftment rates (at Day 28 for neutrophil recover and Day 60 platelet recovery) although this did not impact total Day 100 engraftment percentages. Overall survival at 1 and 3 years was significantly superior with HLA-identical sibling donors versus matched, unrelated donors (78% versus 45%, and 76% versus 39%, resp.). Of the 23 deaths, the most prevalent causes of mortality were infection (31%), graft failure (27%), and interstitial pneumonia (13%). Only one patient died due to GVHD. The use of irradiation, the number of transfusions, time duration between diagnosis and transplant, and age at transplant were not associated with differences in survival. Data regarding iron overload status at time of transplant were not available. 

The poor outcomes of MUD transplantation are likely due to the relatively high number of antigen-based matches involved in these reports. In the relatively more HLA-homogenous Japanese population, nineteen Japanese DBA patients [18] receiving transplants from varying sources (42% sibling matched, 32% matched unrelated, 21% mismatched unrelated, 5% mismatched related) and conditioned with cyclophosphamide plus irradiation in 68% and without irradiation in 32% had (excluding one early death) 100 day neutrophils engraftment rates of 90%, with both nonengrafting patients receiving unrelated, 4/6 cord blood transplants. GVHD prophylaxis was largely cyclosporine-based; incidence of acute GVHD grades II–IV was 25% and only one case of chronic GVHD was reported. Overall survival at 5 years was 79%; 5-year failure-free survival was 100% in recipients of bone marrow transplants compared to 40% of cord-blood recipients. Observable outcome differences between HLA-identical sibling BMT and MUD BMT were limited to recovery time of platelets although overall engraftment rates were not significantly different. Additionally, recent mention of the DBA Registry data reports early overall survival rate of 85% in MUD transplants performed since 2000, far superior to more dated publications [22]. 

Historically, upfront transplantation for DBA was largely restricted to transfusion-requiring patients with available sibling-matched donors. It is now evident that MUD transplantation is an increasingly feasible option although more data and experience are required before identifying its exact role for DBA. Limiting transfusion exposures, controlling iron overload, minimizing steroid-induced adverse effects, and monitoring for malignancy continue to be of crucial importance in the care of these patients.

## 4. Dyskeratosis Congenita

Dyskeratosis congenita (DC) is a failure in telomere maintenance resulting in the classic triad of abnormal skin pigmentation, nail dystrophy, and leukoplakia of the oral mucosa as well as lacrimal duct stenosis, pulmonary fibrosis, and bone marrow failure. Mutations in the telomere maintenance complexes (including DKC1, TERC, TERT, NOP10, NHP2, TINF2) result in shortened telomeres especially in stem cells undergoing high proliferative rates. Abrogation of hematopoietic stem cell replicative potential results in bone marrow failure in 85% of patients and is the cause of death in 80% [23]. Similar to Fanconi Anemia patients, DC patients are at increased risk for hematologic and solid malignancies with a cumulative incidence approaching 50% at 50 years of age [24, 25]. 

Initial HSCT attempts using myeloablative conditioning resulted in high morbidity and mortality, notably from pulmonary fibrosis [26]. Several case reports of successful transplantation using nonmyeloablative regimens have been published [27, 28]. A series of six patients receiving unrelated donor bone marrow (one 8/8, one 7/8), unrelated double cords (4/6 for both cords in one patient, and 4/6 and 5/6 for two other patients), and HLA-identical related peripheral blood stem cells used nonmyeloablative conditioning with cyclophosphamide, fludarabine, alemtuzumab, and 200 cGy TBI [29]. GVHD prophylaxis consisted of cyclosporine and mycophenolate. Five patients engrafted (one patient died prior to engraftment at 1 month); two out of six patients died from infectious complications, two patients developed aGVHD (skin, gut) Grade II–IV, and one patient developed chronic skin GVHD; overall survival was 67% with median follow-up of 2 years. The authors' review of published case reports using non-myeloablative conditioning in transplantation for DC (excluding the authors' six reported patients) yielded 18 patients (11 related bone marrow transplants, 5 unrelated bone marrow transplants, 2 unrelated cord transplants). 91% of related transplant recipients were alive with a median follow-up of two years. 40% of matched-unrelated transplant recipients were alive with a median follow-up of 15 months; there was a high incidence of GVHD in the 60% of patients that died. Transplantation using reduced intensity conditioning is therefore indicated for DC given its high probability of bone marrow failure and death, with expected two year post-transplant survival rates of 40–90% depending upon stem cell source.

## 5. Severe Congenital Neutropenia

Severe congenital neutropenia (SCN) is a constellation of syndromes consisting of arrested myeloid development resulting in neutropenia. The genetic pathology is largely due to mutations in ELA2 and GFI1; a subset of patients have mutations in the Hax1 gene (Kostmann's neutropenia) and rare, congenital X-linked mutations in CSF3R [30]. The natural history of the disease includes infectious complications and, consistent with other bone marrow failure syndromes, an elevated risk of myelodysplastic and leukemic transformation. Approximately 90% of patients respond to GCSF administration with a subsequent decrease in sepsis-related mortality to almost 1% per year during the first decade of life [31]. However, GCSF poor and nonresponders remain at particularly high risk for hematopoietic dysplasia/malignancy with a cumulative incidence of 21% following 10 years of GCSF therapy that was thought to dramatically climb thereafter [31]. However recently updated North American registry data suggest a relative plateau in the hematologic malignancy risk after 10–15 years, similar to that seen in Fanconi Anemia and dyskeratosis congenita [32]. It is thought that the SCN pathology and degree of severity particularly predisposes to malignancy as the incidence of MDS/AML transformation at 10 years was only 15% for patients without neutropenia on GCSF versus 34% for patients with persistent neutrophil counts below 2100/microliter despite high doses (greater than 8 mcg/kg/day) of GCSF. Although it remains controversial if prolonged GCSF therapy predisposes to malignant transformation it is notable that large cohorts of other patients receiving chronic GCSF therapy for cyclic and idiopathic neutropenia have not reported malignant transformation [33]. Furthermore the risk of malignant transformation in SCN patients receiving high doses of GCSF does not appear to be greater than other bone marrow failure syndromes with high malignancy rates such as Fanconi Anemia and dyskeratosis congenita [32].

Zeidler et al. reported 11 SCN patients without malignancy who received HSCT [34]; 8 received sibling-matched transplants (5 BMT, 1 cord, 1 BMT and cord, 1 PBSC) and 10 received myeloablative conditioning with busulfan/cyclophosphamide ± ATG, thiotepa, or melphalan. All patients receiving myeloablative conditioning engrafted regardless of source although 40% of tested patients had graft chimerism (30%–84% donor) at 6–12 months post-transplant. Acute GVHD grade II–IV was present in 2 of the unrelated BMT recipients. Overall survival of the 10 patients receiving myeloablative regimens was 80% at median follow-up of 10 months. The French SCN registry reported HSCT outcomes on 5 SCN patients without malignant transformation. Donors included unrelated cords (2) and unrelated bone marrows (2), and related bone marrow (1). All received myeloablative regimens with 80% engraftment and 40% mortality due to infection at one year post-transplant [35]. In contrast, 18 Japanese patients with SCN without malignant transformation underwent transplantation from sibling-matched (9) or matched-unrelated donors (9) following myeloablative conditioning in 12 cases and non-myeloablative in the remaining 6 patients. Although four patients encountered primary graft failure (including two sibling matched donor transplants) and received a second transplant, 16 of the 18 patients are considered disease-free with a median follow-up of 6 years [36]. 

Hematopoietic stem cell transplantation in SCN patients in the setting of MDS/AML carries high mortality (60%–80% as reported in case series [37] and 1998 SCNIR registry data) but is the only curative option. Choi et al. [37] reported 6 SCN patients with transformation to MDS (2) or AML (4) at a median age of 13 years old; patients received sibling-matched (1), matched-unrelated (3), or single mismatch unrelated (2) donors and were conditioned with busulfan-based (5) or TBI-based (1) regimens. The two MDS patients did not receive induction chemotherapy, engrafted, and were disease-free from 2 to 4 years posttransplant, although both remain on immunosuppression for chronic GVHD. The four AML patients received induction chemotherapy and were transplanted in complete remission; those receiving single-mismatched grafts had primary graft failure. Ultimately all four patients died: two died from chronic GVHD, one died from primary graft failure, and one died from relapsed AML. The French SCN registry [35] reported four SCN patients transplanted for MDS (3) and ALL (1) who received unrelated cords, bone marrows, or related bone marrow after myeloablative conditioning. All four patients engrafted and three survived (one died from septic shock) with median followup of 2 years. One patient required a second transplant for relapsed MDS/AML. Of note, 2 of the 3 MDS patients did not receive induction chemotherapy prior to transplant. 

Transplantation is therefore indicated for patients unresponsive to GCSF and should be strongly considered for mild response to high doses of GCSF given this group's risk of malignancy and the disparity between transplant survival prior to malignant transformation compared to afterwards. The utility of induction chemotherapy is unknown in patients with MDS, as successful donor engraftment and relapse-free survival without induction chemotherapy have been demonstrated [35].

## 6. Shwachman-Diamond Syndrome

Shwachman-Diamond Syndrome (SDS) is an autosomal recessive disorder associated with mutation in the Shwachman Bodian Diamond Syndrome gene, *SBDS, *involved in ribosomal biogenesis and mitotic spindle association. The disorder is classically associated with neutropenia (intermittent or chronic) and exocrine pancreas deficiency. However, patients often have multiple hematopoietic cell lineage involvement with anemia and thrombocytopenia seen in up to 80% of patients [38] and stromal cell abnormalities have also been reported [39]. Other findings include skeletal, cardiac, endocrine, and renal abnormalities. 

Similar to Fanconi Anemia and dyskeratosis congenita, SDS patients have an increased risk for the development of MDS and AML. The French SCN registry recorded a 19% cumulative incidence of MDS/AML by 20 years of age in 55 SDS patients; this incidence increased to 36% by 30 years of age [40]. In contrast, the NCI's IBMF registry of 17 SDS patients followed for a cumulative duration of 274 person-years did not observe any malignancy development [25]; further registry followup with more patients is necessary to more accurately identify SDS patients' true malignancy predisposition. 

Donadieu et al. reported the outcomes of 10 SDS patients receiving HSCT; 5 of the patients were transplanted for bone marrow failure without malignancy [41]. Four received unrelated BMT (either 9/10 or 10/10 HLA-matched) and one received a sibling-matched BMT. Myeloablative conditioning utilized busulfan-based (3) or TBI-based (2) regimens, with 3 patients also receiving ATG. All five patients engrafted and were alive at a median of 8 years with no chronic GVHD grade III-IV. The study also reported five SDS patients transplanted for MDS (4) and leukemic (1) transformation with sibling-matched donors (3) and 9/10 MUD (2) [41]. Myeloablative conditioning was with busulfan (3) or TBI (2) regimens. Only one patient received induction chemotherapy. Four patients died; two died of sepsis and organ toxicity prior to 40 days posttransplant, one patient relapsed at 5 months, and one died of respiratory complications at 1.7 years post-transplant. Other myeloablative regimens have reported high transplant-related mortality with significantly elevated transplant-related cardiac toxicity [42]. A review of 27 case-reported SDS patients transplanted using myeloablative conditioning demonstrated a 18% mortality in patients transplanted for cytopenias(s) versus a 53% mortality rate in patients with active dysplasia/malignancy [43].

Reduced-intensity conditioning has been successfully used in SDS patients. Sauer et al. used a conditioning regimen of fludarabine, treosulfan, and melphalan in three recipients transplanted for pancytopenia (2) and MDS (1) [44]. Two patients were alive at 16 and 24 months post-transplant without respiratory complications; one patient transplanted for pancytopenia died at Day +98 due to idiopathic pneumonia syndrome. Bhatla et al. reported seven transplanted patients conditioned with alemtuzumab, fludarabine, and melphalan in varying states of hypocellularity [45]. One patient was transplanted for AML postinduction chemotherapy, another for MDS. 57% received sibling-matched marrow stem cells; the remaining three patients received unrelated peripheral blood or bone marrow stem cells. All 7 patients engrafted and were alive with median followup of 1.5 years. There was no acute GVHD Grade III-IV using prophylactic treatment with cyclosporine and methotrexate/steroids (depending on stem cell source).

Transplantation in SDS patients with active dysplasia or malignancy is associated with significantly higher mortality. The risk of malignancy is elevated although to what degree is unclear. Therefore, close surveillance of these patients is advised for early detection of worsening cytopenias with clonal evolution. Reduced-intensity conditioning regimens have been successfully used in limited series with good engraftment rates and decreased transplant-related mortality.

## 7. Conclusions

The bone marrow failure syndromes constitute a heterogeneous group of molecular pathologies that share a phenotype of hematopoietic disruption. Hematopoietic stem cell transplantation remains the only curative treatment option at present but the indications for transplant are dependent upon the predisposition to severe bone marrow failure/malignancy transformation and must be balanced against the known increased transplant-related complication rates seen in such patients. Furthermore, it is important for physicians to recognize the potential for underlying BMFS in patients, regardless of age, presenting with aplasia and to tailor the diagnostic workup and potential transplantation regimen accordingly. Of note, BMFS-associated aplastic anemia does not respond to the immunosuppressive therapy regimens used for acquired aplastic anemia. Therefore time should not be wasted with such treatments as subsequent malignant transformation may result in severely increased transplantation-related mortality, most notably reported in patients with severe congenital neutropenia and Shwachman-Diamond syndrome. In addition, patients with Fanconi's Anemia and dyskeratosis congenita require reduced intensity conditioning regimens that likely hinder transplantation survival in the setting of malignancy.

It is imperative that BMFS patients are entered into their respective registries. These databases enable these rare patients to be studied in a systematic fashion for both immediate and long-term sequelae. Late transplant-related effects may be seen in nearly any organ system, with notable risks for cardiac, pulmonary, renal [46], endocrine, psychiatric, and fertility dysfunction [47]. Limited data on survivors of pediatric HSCT report “good” quality of life with median Lansky/Karnofsky performance scores of 90 [48]. Furthermore, transplant survivors of nonmalignant disorders may enjoy a superior health-related quality of life relative to survivors of malignant disorders [49]. However, given the known increased incidence in transplant-related morbidity as well as nonhematopoietic complications in BMFS patients it will be important for the registries to continue their long-term followup.
